# Tree cover mapping based on Sentinel-2 images demonstrate high thematic accuracy in Europe

**DOI:** 10.1016/j.jag.2019.101947

**Published:** 2020-02

**Authors:** Thor-Bjørn Ottosen, Geoffrey Petch, Mary Hanson, Carsten A. Skjøth

**Affiliations:** School of Science and The Environment, University of Worcester, Worcester, UK

**Keywords:** Sentinel-2, Band combinations, Tree cover, Unsupervised classification, Copernicus

## Abstract

The spatial and temporal distribution of trees has a large impact on human health and the environment through contributions to important climate mechanisms as well as commercial, recreational and social activities in society. A range of tree mapping methodologies has been presented in the literature, but tree cover estimates still differ widely between the individual datasets, and comparisons of the thematic accuracy of the resulting tree maps are rather scarce. The Copernicus Sentinel-2 satellites, which were launched in 2015 and 2017, have a combination of high spatial and temporal resolution. Given that this is a new satellite, a substantial amount of research on development of tree mapping algorithms as well as accuracy assessment of said algorithms have to be done in the years to come. To contribute to this process, a tree map produced through unsupervised classification was created for six Sentinel-2 tiles. The agreement between the tree map and the corresponding national forest inventory, as a function of the band combination chosen, was analysed and the thematic accuracy was assessed for two out of the six tiles. The results show that the highest agreement between the present tree map and the national forest inventory was found for bands 2, 3, 6 and 12. The present tree map has a relative difference in tree cover between 8% and 79% compared to previous estimates, but results are characterised by large scatter. Lastly, it is shown that the overall thematic accuracy of the present map is up to 90%, with the user’s accuracy ranging from 34.85% to 92.10%, and the producer’s accuracy ranging from 23.80% to 97.60% for the various thematic classes. This demonstrates that tree maps with high thematic accuracy can be produced from Sentinel-2. In the future the thematic accuracy can be increased even more through the use of temporal averaging in the mapping procedure, which will enable an accurate estimate of the European tree cover.

## Highlights

• A tree map was created using unsupervised classification for six Sentinel-2 tiles.
• The combination with highest agreement with the NFI was the bands 2, 3, 6 and 12.
• Good qualitative agreement between the present map and the NFI.
• The map shows between 8% and 79% greater tree cover compared to previous estimates.
• The overall accuracy of the present map was assessed to be up to 90%.

## 1 Introduction

Trees serve as a major carbon pool contributing to important feedback mechanisms to the earth’s climate (Bonan, 2008). Likewise, trees are known to release gases, such as Biogenic Volatile Organic Compounds (BVOC) (Kesselmeier and Staudt, 1999), and Primary Biological Aerosols (PBA), such as pollen (Pauling et al., 2012) and fungal spores (Sadys et al., 2014), to the atmosphere. Repeatedly, it has been demonstrated that location and abundance of trees are important in relation to the release of VOC (Arneth et al., 2011; van Meeningen et al., 2016) and their contribution towards production of secondary organic aerosols (Oderbolz et al., 2013; Tchepel et al., 2014) or PBA (Hernandez-Ceballos et al., 2011; Pauling et al., 2012). Furthermore, the spatial and temporal distribution of trees is known to be important for commercial, recreational and social activities in society (FAO, 2015) as well as the ecological or biodiversity functionality of the landscape (e.g. Ren et al., 2013; Schindler et al., 2013). It is thus evident that the spatial distribution of trees and changes in the spatial distribution of trees over time has a large impact on human health and the environment.

A range of tree mapping methodologies has been presented in the literature. Focus in this section will be on mapping of trees in the United Kingdom (UK) due to the scarcity of map comparisons for other countries. Skjøth et al. (2015) assessed the accuracy of Corine Land Cover (Bossard et al., 1994) and Globcover (Bicheron et al., 2009) against the National Forest Inventory (NFI) over the UK (Forestry Commission, 2001). Despite reported high thematic accuracy for Corine Land Cover (Büttner and Maucha, 2006; Caetano et al., 2006) and Globcover (Defourny et al., 2009), large biases were found in these compared to the national dataset. Similar map comparison exercises have been carried out at European scale (Seebach et al., 2011a, b). In old cultural landscapes, like the UK, many trees are located in smaller patches, such as hedgerows, or in urban areas (McInnes et al., 2017). A remote sensing approach was used in Kempeneers et al. (2011), who mapped European scale tree cover as presence/absence, and Hansen et al. (2013), who mapped global tree cover as a percentage, both with a spatial resolution of 25 m – 30 m. An estimate by the authors shows a relative difference of 26% in the area of the total UK tree cover between Hansen et al. (2013); Kempeneers et al. (2011) and Forestry Commission (2011), which indicates a considerable uncertainty relating to the total tree cover in the UK. Moreover, Hansen et al. (2013) does not distinguish between broadleaved and coniferous trees, a minimum requirement in a range of scientific applications such as air quality modelling (Oderbolz et al., 2013; Steinbrecher et al., 2009), dynamic vegetation modelling (Hickler et al., 2012), the generation of national forest inventories (Paivinen et al., 2009) and modelling of climate change including future woodland changes (e.g. Jones et al., 2009). As seen, there is a general lack of consensus on mapping methodologies (Hansen and Loveland, 2012) and comparisons of the thematic accuracy of the resulting tree maps are rather scarce.

The Copernicus Sentinel-2 satellites, which were launched in 2015 and 2017, have four bands with a spatial resolution of 10 m and a total band combination of 13 bands, with spatial resolutions ranging from 10 m to 60 m, specifically designed for vegetation monitoring (Drusch et al., 2012). The high spatial, temporal and radiometric resolution of data from this satellite should enable the creation of tree cover maps with a higher thematic accuracy than previously achieved, and recent examples include Grabska et al. (2019) and Korhonen et al. (2017). Given that this is a new satellite, a substantial amount of research on development of tree mapping algorithms as well as accuracy assessment of said algorithms have to be done in the years to come. To contribute to this process, a tree map for six selected Sentinel-2 tiles was created, the optimal choice of spectral bands as input to the map was analysed, and the accuracy of this map was assessed.

Section 2.1 describes the creation of the tree map using unsupervised classification. The data included in the respective analyses is specified in Sections 2.2, Section 2.3 describes the analysis of the optimal choice of spectral bands and Section 2.4 details how the accuracy assessment was performed. The study area is described in Section 2.5 as a foundation for the discussion of the map accuracy. The results and discussion are presented in Section 3 and the conclusion in Section 4.

## 2 Methods

### 2.1 Tree cover mapping

In order to support monitoring processes, reduce the cost of the development and increase the production speed, the mapping methodology should proceed without analyst interference (Hansen and Loveland, 2012). The tree map in the present study was therefore created using an unsupervised classification approach. The tree mapping algorithm consisted of a number of steps:

1. Removing pixels with cloud cover, defect pixels, no-data pixels, and saturated pixels using the accompanying masks for the individual Sentinel scene.
2. Resampling all bands to 10 m × 10 m using nearest neighbour interpolation.
3. Normalizing the bands using mean centring and division by the standard deviation to remove effects of different scale of reflectance in the images obtained in the different bands following the approach of e.g. Nguyen et al. (2018). Tests showed that the accuracy of the mapping procedure increased considerably through adding this step.
4. Classification of the satellite image using unsupervised *k*-means classification within R. The *k*-means algorithm in R was very time consuming on the 5 GB tiles from Sentinel-2. The approach was therefore improved numerically by using Intel® Data Analytics Acceleration Library (DAAL) (https://software.intel.com/en-us/intel-daal) linked directly within R. The unsupervised classification was performed with 25 classes, based on the authors experience with similar classification exercises, and with a maximum of 20 iterations to limit calculation time. This number is higher than previous identified optimum number of 12 classes in specific Landsat scenes (Yıldırım, 2014) and therefore ensures sufficient number of classes without compromising quality. Sensitivity tests showed that the mapping algorithm was not particularly sensitive to these choices.
5. The classified image was filtered to remove non-vegetation pixels by calculating the Normalized Difference Vegetation Index (NDVI) (Tucker, 1979) for the entire image by using band number 4 (red) and 8 (Near-Infrared) and setting a lower threshold. The lower NDVI threshold for vegetation was found by analysing the distribution of NDVI values in the image that was mapped as forest in Corine Land Cover. The assumption was that pixels with an NDVI less than the median minus the distance from the median to the 95-percentile of the distribution were non-vegetation pixels (e.g. buildings, roads or lakes found in forests), that according to the definition can be expected to be present in forested areas identified by the Corine Land Cover. The removal of these pixels also has the effect of removing clouds, shadows and other artefacts not included in the accompanying mask files.
6. The classes belonging to respectively coniferous and broadleaved trees were labelled using the forests classes from Corine Land Cover as training data but with error pixels and non-vegetation removed (step 1 and step 5). Broadleaved and coniferous forests in Corine Land Cover can contain up to 25% of other land cover types. Moreover, Corine Land Cover has a minimum mapping unit of 25 ha (Bossard et al., 1994). These two properties introduce noise in the training data. To circumvent this problem, an iterative procedure, with the aim of finding the dominating classes, from the classification performed in step 4, for respectively broadleaved and coniferous forests, was developed:
  a. Within each Sentinel-2 scene, the polygons for respectively coniferous forest and broadleaved forest from Corine Land Cover were sorted in descending order as a function of the area. The iterations proceeded from the largest polygons to the smallest based on the assumption that the uncertainty was largest on the smallest polygons in Corine Land Cover, an assumption that was confirmed during the algorithm development phase.
  b. The largest polygon for broadleaved and coniferous trees was then masked out from the classified image (step 4) after the filtering (step 5) and the proportion of pixels in the respective classes defined by the *k*-means algorithm was calculated separately for broadleaved trees and coniferous trees.
  c. This procedure was then repeated for the second largest polygon for respectively broadleaved and coniferous, and the pixels from the new polygon added to the distribution created in step b).
  d. Convergence was checked by comparing the percentage change in each class in the distribution between iterations, and convergence was reached when the largest change in a class was less than 1%. If convergence was not reached step c) was repeated with the next largest polygon until convergence. As the polygons are getting smaller and smaller, convergence will eventually be achieved in this way.
  e. All 25 classes from the *k*-means algorithm applied on the entire image and extracted within the Corine Land Cover forest areas without non-vegetation classes were then labelled as either *mostly broadleaved* or *mostly coniferous* trees based on which category had the largest proportion of the selected class.
  f. Subsequently, for respectively the *mostly broadleaved* classes and the *mostly coniferous* classe*s,* a *k*-means clustering was applied to divide the distribution into two classes: Dominating and non-dominating. This resulted in a subset of the 25 classes where the forest type could be identified.
  g. The dominating classes were then labelled as either broadleaved or coniferous forest. The remaining classes were labelled non-forest and the separation of the 25 classes into three categories was applied on the entire image.

In this way, a tree map was created without analyst interference. To test the sensitivity of the method to the use of Corine Land Cover as training data, the tile 30UWC from 19.07.2016 was classified using Globcover (Bicheron et al., 2009) as training and the results compared with the result using Corine Land Cover. The details of replacing Corine Land Cover with Globcover and the results are described in Appendix A.

### 2.2 Data

Six Sentinel-2 single tile images were downloaded as L1C data from United States Geological Survey (USGS) earthexplorer (https://earthexplorer.usgs.gov/) for the creation of a tree map. The level 1C processing includes radiometric and geometric correction using ground control points and a digital elevation model to correct for parallax error (Drusch et al., 2012). L1C data provide top of atmosphere reflectances, and thus no further preprocessing was applied to the images. The tiles were selected to cover the summer period (June-August) 2016 and to have as small a cloud cover as possible. The tile 30UWC was selected since it covers Worcester, UK an area familiar to the authors. Two images of this tile were downloaded to elucidate seasonal differences. The tile 30VUH was selected to cover an area of Scotland, which has a much larger fraction of coniferous trees compared with most of England and thus provides a different type of landscape to the analysis. The tile 32VNH covers an area in western Denmark and the tile 33VUC covers an area in eastern Denmark and southern Sweden. These were selected since high resolution tree cover maps (www.kortforsyningen.dk, www.lantmateriet.se) used by national forest inventories (Nord-Larsen et al., 2016) were available for these countries for the testing of band combinations (described in Section 2.3) and because the areas are familiar to the authors. The available data is here available as final classified data sets delivered in the form of shape files, where the central input data for providing the tree cover maps in all regions are based on a combination of high-resolution aerial photography and administrative records combined with sites visits all with a spatial accuracy much higher than the 10 m resolution provided by Sentinel-2.

The tile 30TWN was selected as a blind test of the forest mapping methodology in Southern Europe, since tile 30UWC was used during the development of the algorithm. The tile covers an area in Northern Spain selected to both have a large urban fraction and substantial tree cover, to allow the accuracy assessment using Google Earth. The algorithm was applied to one image at a time, to better analyse the performance of the algorithm, to keep the data and calculation requirements small for the present study, and to limit the study scope. Future work should aim at analysing the impact of the input data on the accuracy of this algorithm as well as related algorithms. Sentinel-2 provides a new opportunity for methods development in land cover analysis by providing a large number of images over the same area taken within a short time span. This enables new possibilities for land cover analysis and the associated error assessment by taking into account multiple images within the area of interest. Such improvements are likely to remove the occasional errors caused by outliers in the data set, thereby increasing the accuracy of the final map. Red, green, blue images of the Sentinel-2 scenes can be seen in Fig. 1 in the supplementary material, the location of the individual tiles can be seen in Fig. 2 in the supplementary material and the properties of the individual tiles are summarized in Table 1.

**Table 1:** Properties of the Sentinel-2 tiles used in the present study.

Tile code:	Location:	Date:	Cloud Cover (%):	Solar Zenith Angle (°)
30UWC	Worcester, UK	19.07.2016	0.03	32.5
30UWC	Worcester, UK	15.08.2016	0.61	39.7
30VUH	Scotland	24.08.2016	6.48	46.1
32VNH	West Denmark	24.07.2016	4.89	37.9
33VUC	East Denmark and Southern Sweden	24.07.2016	1.62	37.4
30TWN	Spain	16.07.2016	2.53	25.9

### 2.3 Testing band combinations

To determine whether all 13 bands from the Sentinel-2 satellite were needed in the algorithm described in Section 2.1, or whether some bands made the classification noisier, the algorithm was run for all band combinations of 3 to 13 bands. To avoid subjective assessments of which bands to include and which to leave out, all 13 bands were included in this part of the analysis. This was done for the five Northern European images due to the availability of recent high resolution tree cover maps as described in Section 2.2. This summed to a total of 8100 combinations. For each classification the wall-to-wall kappa coefficient (Cohen, 1960; Congalton et al., 1983) between the national forest inventory and the tree map was calculated. The kappa coefficient is a popular approach to map comparison in remote sensing (Foody, 2006), since a visual comparison of the National Forest Inventories with the red, green, blue image of the corresponding satellite image showed that these also contained errors. The kappa coefficient should not be used for accuracy assessment (Pontius and Millones, 2011) (the details of this analysis are described in Section 2.4) but can be used to assess “inter-rater agreement” (Foody et al., 2013). This choice is also based on that the present study only analyses the difference in kappa coefficient for the respective band combinations, which removes the risk associated with using one specific kappa coefficient.

The satellite-based tree map was filtered to remove small patches of trees before the calculation of the kappa coefficient to make it comparable with the corresponding national forest inventory. This resulted in a minimum mapping unit of 0.5 ha for images 30 UWC and 30VUH, 0.25 ha for 32VNH and 0.01 ha for 33VUC, since the Swedish data are made in a way that does not operate with a minimum mapping unit. The kappa coefficients were summed across the five images, different approaches to select an optimal (based on the images in the present analysis) band combination were explored and an optimal band combination, conditional on the present algorithm and input data, was chosen to produce an automated tree cover map using Sentinel-2.

### 2.4 Accuracy assessment of forest map

The accuracy of the map resulting from the analysis described in Section 2.1 was assessed at tiles 30UWC and 30TWN to cover both Northern and Southern Europe. No filtering was applied to the map in this part of the analysis, and the minimum mapping unit is therefore 0.01 ha. The accuracy assessment needed reference data which were derived from Google Earth as described in Section 2.4.2. High resolution images from Google Earth are available for the entire 30UWC tile and areas close to the larger cities for the 30TWN tile. Reference data points therefore cover the entire tile 30UWC and within 10 km of the four cities Bilbao, Vitoria, Logrono and Pamplona in tile 30TWN. The accuracy assessment for both images followed the sampling design, response design and analysis methodology of Stehman and Czaplewski (1998).

#### 2.4.1 Sampling design

To test the thematic accuracy of the map, an accuracy assessment dataset was produced. To generate this dataset, 999 pixels were extracted from the image. The sampling was made using stratified (broadleaved trees, coniferous trees and no trees) random sampling (Stehman, 2009) with equal sample size for each stratum, since the area covered by the no trees category naturally will be much larger than the area covered by the two forest categories for both images. This ensured 333 pixels in each stratum, which exceeds the 100 pixel threshold, which according to Stehman (2001), is required to obtain a standard error of 0.05 on the overall accuracy almost regardless of the sample size.

#### 2.4.2 Response design

Stehman and Wickham (2011) discuss the use of pixels, blocks of pixels and polygons as the spatial unit for accuracy assessment based on the recommendations in Congalton and Green (2009). They show, through a numerical example, that the effect of moving from pixels to blocks of pixels to polygons has a small effect on the overall accuracy of the map. It was therefore decided to stick with 10 m × 10 m pixels as the spatial unit for the accuracy assessment, an approach also used by e.g. Feng et al. (2016) and Wickham et al. (2017).

Each 10 m × 10 m pixel was assigned a primary land cover class and eventually a minor land cover class if this was present following similar approaches as Benza et al. (2016); Shubho et al. (2015); Wickham et al. (2017); Yan and Roy (2016).

The collection of reference labels was done by three interpreters within the study group. To enhance consistency among interpreters, a written guide to the classification procedure was produced and 99 points, selected using the sampling design described in Section 2.4.1, for both the tiles 30UWC and 30TWN, were classified by all interpreters. The interpreter did not have access to the forest map from the satellite during classification to avoid biasing the manual classification (blind interpretation). Each interpreter was supplied a Google Earth KML file containing the sample pixels for overlay on Google Earth imagery. The interpreter selected the Google Earth image with an image date as close as possible to the date of the satellite image and with good visibility and subsequently decided the most appropriate land cover category. The interpreter could select among the three categories from the tree map plus “unclassified trees” and “unclassified” for images and points where a distinct category could not be determined. Pixels in the last two categories were subsequently excluded from the analysis and the initial number of 333 sampling points in each category thereby ensured that the total number of pixels is substantially above the minimum number of 100 according to Stehman (2001). The number of remaining pixels can be found in the Results section (Section 3.3).

#### 2.4.3 Analysis

The reference dataset based on Google Earth was used to produce a confusion matrix for the two classified Sentinel-2 images covering three classes (broadleaved trees, coniferous trees and non-trees) and two classes (trees and no trees), by merging the two tree-classes to one. Following recommended “good practice” in accuracy assessment (Olofsson et al., 2014; Stehman and Foody, 2019), the error matrix was reported in terms of estimated area proportions ${\hat{p}}_{ij}$:

(1) $${\hat{p}}_{ij}=W_{i}\frac{n_{ij}}{n_{i+}}$$

Where W_i_ is the proportion of area mapped as class i, n_ij_ is the sample counts of pixels mapped as class i which belong to class j, and n_i+_ is the sample size from stratum i. The user’s accuracy, producer’s accuracy, overall accuracy, plus the proportion of area in each class based on the reference classification along with their corresponding standard errors were calculated using the formulas from Olofsson et al. (2014); Stehman and Foody (2019). The confusion matrix for the two-class case was made using the indicator functions described in Stehman (2014).

### 2.5 Study area

**Fig. 1:**
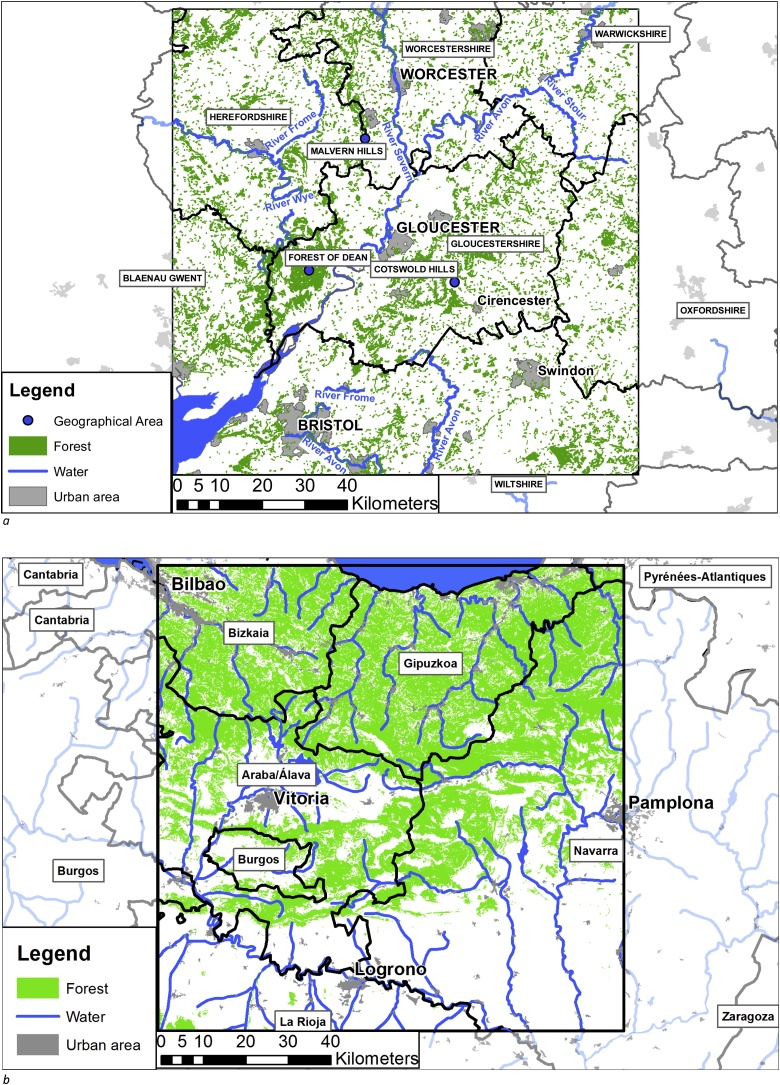
(a) Map of the study area in the North. Data sources: Counties, Urban areas, Geographical areas, rivers (https://www.ordnancesurvey.co.uk/business-and-government/products/strategi.html), Surface water (Corine Land Cover), Forest areas (Morton et al., 2011). The forest polygons with an area < 1.5 ha have been filtered away to increase map readability. Map is produced by the authors. (b) Map of the study area in the south. Data sources: Counties (Eurostat NUTS, https://ec.europa.eu/eurostat/web/gisco/geodata/reference-data/administrative-units-statistical-units/nuts), Urban areas (Bossard et al., 1994) (data from Corine Land Cover 2012), rivers and surface water (Digital Chart of the World, http://www.soest.hawaii.edu/wessel/dcw/), Forest areas (Hansen et al., 2013) reclassified with forests containing more than 50% trees. The forest polygons with an area < 1.5 ha have been filtered away to increase map readability. Map is produced by the authors.

The study area in the North with reference data, tile 30UWC, is centred on the city of Gloucester (Fig. 1a), encompassing Gloucestershire and parts of 9 other counties located in the Midlands, England. The relief of the landscape is marked by the Severn Valley in the centre and associated tributaries with a uniform low level terrain between Gloucester and Worcester and the Bristol Channel to the Southwest (e.g. Sadys et al., 2014). No large upland areas occur within the area, but the land rises towards the Birmingham plateau in the north and towards the massifs of mid-Wales in the west. Nevertheless some prominent hills exist; the Malverns (peak height 425 m), Bredon Hill (293 m), the Cotswold range (up to 300 m) and the Black Mountain (550 m) as seen in Fig. 3 in the supplementary material. The area has one large woodland in the Forest of Dean (Forestry Commission, 2011) and numerous small woodlands and groups of trees (Forestry Commission, 2017; Skjøth et al., 2015), distributed approximately homogeneously across the area and located in both the rural and urban areas. According to Forestry Commission (2011) the area covered by forests amounts to 8.32%. The area is dominated by privately owned woodlands, where broadleaved trees are the most abundant tree type (Forestry Commission, 2002). The broadleaved part is typically dominated by *Quercus sp*, *Fraxinus sp* and *Fagus sp*, while the coniferous part often consists of a broad range of unclassified species complemented by *Picea abies* and *Pinus Sylvestris* (Skjøth et al., 2008). The rest of the landscape covers urban areas and in particular agricultural areas used for annual crops within rotation systems and permanent pastures (Sadyś et al., 2015), but also with significant areas for fruit production (e.g. Sadys et al., 2014). The climate of the region is relatively uniform and characterized as maritime and cold temperate (UK Met Office, n.d.-a) with mild winters and warm summers, an annual mean temperature around 10 degrees, and regular rainfall throughout the year ranging from about 600 mm/year to more than 800 mm/year (e.g. Sadys et al., 2014; UK Met Office, n.d.-b).

The study area in the South with reference data, tile 30TWN, is bordered by the cities of San Sebastian, Bilbao, Logrono and Pamplona (Fig. 1b). The region encompasses the three regions of Gipuzkoa, Vizcaya, and La Rioja and partly covers several other regions, located in the most Northern parts of Spain towards the Bay of Biscay. The central part of the region is covered by the Cantabrian Mountains with elevation up to 1500 m (as seen in Fig. 4 in the supplementary material), contrasted by the large Ebro Valley and the Ebro River in the southern part of the domain. The area has numerous larger woodlands, in particular in the mountainous part but also in lower areas to the North, while the valleys such as the Ebro Valley are mainly covered by agricultural land, therefore containing very few trees. The total tree cover of the region is, according to Hansen et al. (2013), 41.8%. The coniferous part of the woodland is dominated by various types of pinus species such as *Pinus sylvestris*, *Pinus halepensis* and *Pinus nigra*, while the broadleaved part is dominated by *Fagus sylvatica* and several *Quercus* species such *Quercus ilex*, *Quercus robur* and *Quercus faginea* (Skjøth et al., 2008). The climate of the region varies substantially due to the large variations in elevation and is, according to generalised maps for the global climate (UK Met Office, n.d.-a), in a region partly covered by temperate and partly by Mediterranean climate. This means that it is a region where winters tend to be warm and wet while summers are dry with little or no rainfall, here considerably modified by the presence of mountains. This has the effect that the annual average rainfall in the region can be below 400 mm/year or above 700 mm/year and that mean annual temperatures can be higher than 15 degrees Celsius in the Ebro Valley and lower than 12 degrees in the nearby elevated terrain (e.g. Vicente-Serrano et al., 2003).

## 3 Results and discussion

### 3.1 Testing band combinations

**Fig. 2:**
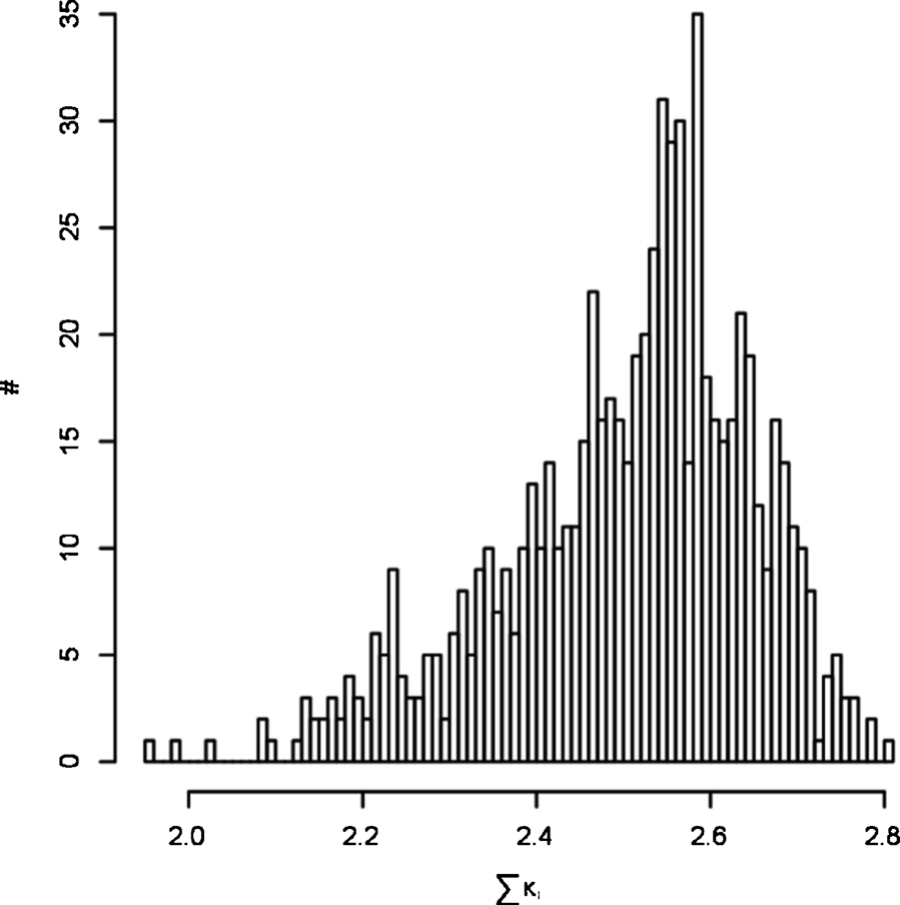
Histogram of sum of kappa coefficients across all five images for all band combinations with n = 4.

The calculation of the wall-to-wall kappa coefficient with the corresponding national forest inventory for all 8100 band combinations shows that the highest summed kappa coefficients generally are 2.7 to 2.8, where the theoretical maximum is 5.0 and the highest kappa-coefficient is found when using a combination of four bands (Table 2). Typically, the coefficients vary from 2 to 2.8, where the highest abundance is in the range 2.4–2.6 as seen in Fig. 2, which displays the kappa-coefficients for the images applying combinations of four bands. Similar results were obtained for band combinations of other lengths. It is evident that there is a very large scatter between the band combinations with some having very high kappa coefficients and others having very low kappa coefficients. This means, that the driver of the mapping performance with respect to identifying forests in the five examples is not the number of bands, but the choice of bands.

**Table 2:** Combinations with the highest summed κ as a function of number of bands (#). ${\text{κ}}_{\text{i}}$ is the kappa coefficient for image i. The maximum value of $\sum {\text{κ}}_{\text{i}}$ is 5.000 (1.000 for each of the five images). Columns 2 and 3 are respectively the minimum and maximum difference in κ between the best performing combination across all five images and the best performing combination for the individual image for the same n.

#	$\sum \kappa_{i}$	Min$\left(\kappa_{\text{m}\text{a}\text{x}}-\kappa \right)$	Max$\left(\kappa_{\text{m}\text{a}\text{x}}-\kappa \right)$	Combination:
3	2.791	0.012	0.068	2, 5, 6
4	2.803	0.010	0.042	2, 3, 6, 12
5	2.799	0.014	0.042	3, 5, 6, 11, 12
6	2.797	0.006	0.050	2, 3, 4, 5, 6, 11
7	2.800	0.016	0.041	1, 3, 4, 5, 6, 11, 12
8	2.784	0.011	0.055	2, 5, 6, 7, 8a, 9, 11, 12,
9	2.787	0.005	0.052	3, 4, 5, 6, 7, 8a 9, 11, 12
10	2.740	0.008	0.062	1, 3, 4, 5, 6, 7, 8a 9, 11, 12
11	2.738	0.012	0.071	1, 2, 3, 5, 6, 7, 8, 9, 10, 11, 12
12	2.734	0.019	0.103	1, 2, 3, 4, 5, 6, 8, 8a 9, 10, 11, 12
13	2.680	0.021	0.072	1, 2, 3, 4, 5, 6, 7, 8, 8a, 9, 10, 11, 12

The maximum kappa coefficients using combinations of between four and seven bands are almost equal. It is evident that bands 2, 3, 6 and 12 appear in many of the combinations. Band 2 is the blue band (496.6 nm, 10 m), band 3 is the green band (560.0 nm, 10 m), band 6 is a red-edge band (740.2 nm, 20 m), and band 12 is a short-wave infrared band (2202.4 nm, 20 m) and this combination is also the highest scoring combination of all bands (Table 2). Using USGS Spectral Characteristics Viewer (https://landsat.usgs.gov/spectral-characteristics-viewer), it can be seen that these bands are particularly suitable to separate different types of vegetation. It is natural that band 4 and band 8 will not contribute much to the classification, since these two bands are already included in the analysis through the NDVI-filter. Columns two and three in Table 2 show that the difference between the individual combinations’ performance in each image is larger than the difference between the performances of the individual combination, which indicates that the highest agreement is achieved by a different band combination for each of the Sentinel images. This result is also seen in Fig. 2, where up to 35 band combinations have a performance differing by less than 1%. This makes it difficult to choose the optimum band combination.

As a way to overcome this problem, the band combinations within 5% of the best performing combination for each image were selected, and the band combinations appearing in the top 5% for all the images are tabulated in Table 3. It is again evident, that the bands 2, 3, 6 and 12 appear in many of the combinations.

**Table 3:** Combinations appearing in the top 5% of each image.

$\sum \kappa_{i}$	Combination
2.755	1, 2, 3, 4, 5, 7, 9, 12
2.803	2, 3, 6, 12
2.774	1, 3, 5, 6, 12
2.771	1, 2, 3, 5, 6, 12
2.800	1, 3, 4, 5, 6, 11, 12
2.797	2, 4, 5, 6, 12
2.799	3, 5, 6, 11, 12
2.797	2, 3, 4, 5, 6, 11
2.757	3, 4, 5, 6, 7, 9, 11, 12

As can be seen from Table 3, the best performing combination with four bands, which is also the best performing combination for the entire dataset, is among the top 5% combinations for each image. It is therefore selected for the following accuracy assessment. Given that this analysis shows a negligible small difference in agreement with the NFIs between the selected band combination and a large number of other band combinations, this choice of band combination must be considered a provisional result. Nevertheless, the results clearly illustrate that using all available bands in Sentinel-2 for this type of land cover analysis does not provide the best results. Future work should aim at arriving at a more definitive answer to the question of choice of bands e.g. through performing this analysis on a larger and more variable set of Sentinel-2 tiles.

### 3.2 Tree mapping

**Fig. 3:**
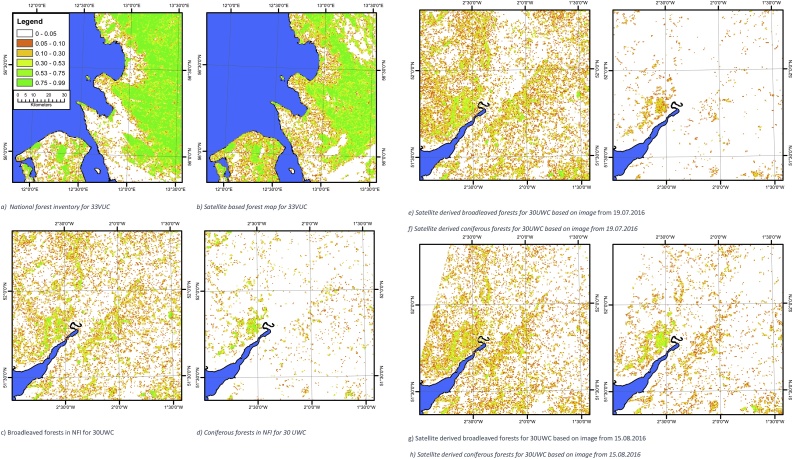

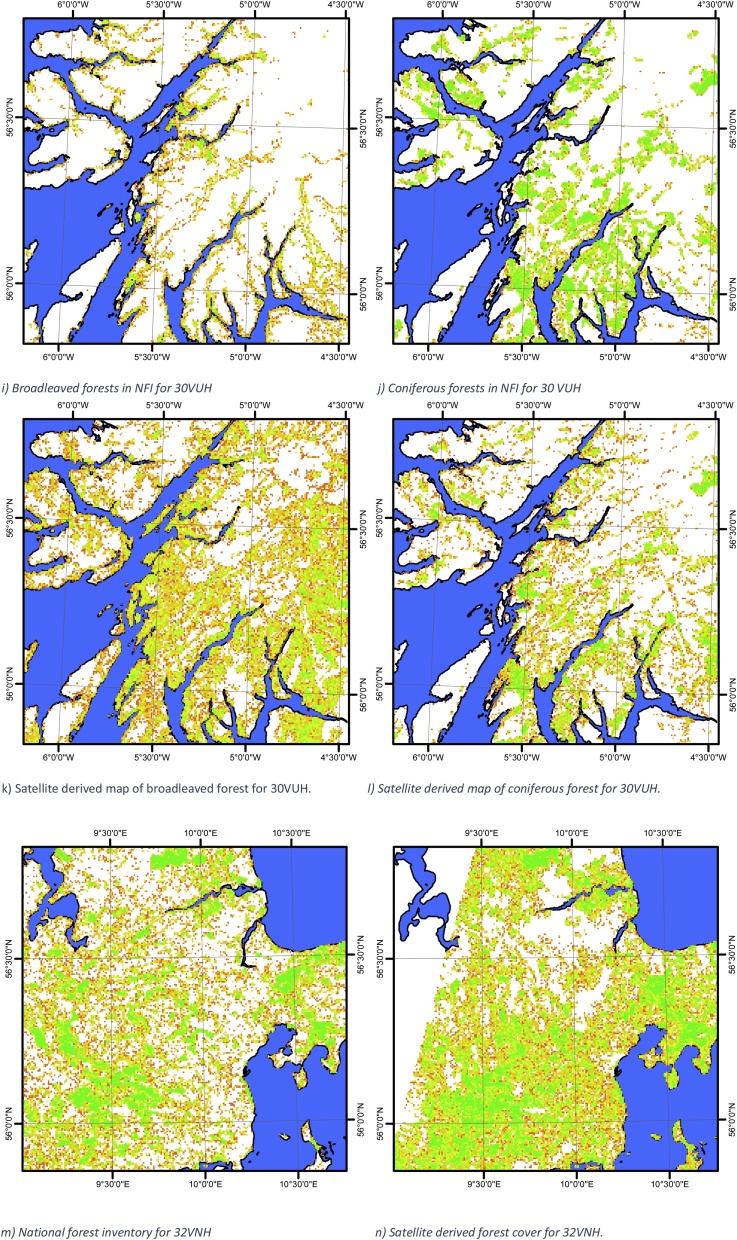
Maps of the fractional cover of respectively the forest/non-forest or broadleaved or coniferous forest either as a satellite based map or based on the corresponding national forest inventory. All maps use the same legend as Fig. 3a, and all maps are aggregated to 500 m x 500 m. The sea is marked with blue. (For interpretation of the references to colour in this figure legend, the reader is referred to the web version of this article).

Maps of the tree cover or broadleaved trees or coniferous trees based on the satellite or the respective national forest inventory are shown in Fig. 3a–n. A visual inspection of the raw data reveals a number of interesting features. For tile 33VUC there is generally good agreement between the NFI and the satellite derived map in areas with a high forest density. On the righthand side of Fig. 3b there are a number of white areas caused by clouds in the satellite image. In the lower left part of the figure and centrally in the picture, the satellite derived map predicts more trees than the NFI. These areas include, according to Corine Land Cover, large amounts of urban residential areas (Corine Land Cover code 112) and sport & leisure facilities (Corine Land Cover code 142), where the latter has actually vast areas covered by summer houses. Local knowledge by the authors establishes the fact that in particular the summer house areas contain large amounts of trees. However, from a land cover perspective these areas are not forests and do therefore not appear in either the national forest inventories or land cover data sets like Corine Land Cover. Nevertheless, these areas contribute substantially to the tree cover in these regions. A secondary effect in this region is minor woodlands and hedges found throughout the part of the region that is designated as agricultural landscape found in both Denmark and Sweden. In this case these minor woodlands are not found in the national forest inventory or the Corine Land Cover. It is therefore a potential source of error, if the Corine classes are used as a training element as the classes are known to be neither spectrally pure or unique e.g. Pekkarinen et al. (2009). We have here solved that issue by two steps: 1) filtering the Corine classes by removing the part of the area that causes the problems with spectral confusion and 2) by using only the most pure fraction of the entire data set as training elements. Some of the difference can also be explained by the algorithm confusing the spectral signal from trees and other types of land cover (e.g. green fields). However, this effect is considered to be of minor importance compared to the very large tree cover found in urban areas, residential areas (summer houses) and the agricultural landscape.

The tile 30UWC (Fig. 3c–f) generally shows good agreement between the NFI and the satellite derived map in the areas with a high woodland density. However, the satellite based map shows a higher amount of broadleaved trees in areas with low tree density (Fig. 3c and e) and a slightly smaller amount of conifers in the picture from 19.07.2016 (Fig. 3f) compared to the picture from 15.08.2016 (Fig. 3h), where the last picture has the closest resemblance to the national forest inventory (Fig. 3d). This suggests that it will be an advantage to take several scenes into account over the same area if the purpose is to create very accurate inventories by using Sentinel-2 images. The higher amount of broadleaved trees could be related to orchards as the area is well known for its cider production. Orchards are technically considered a part of the agricultural landscape and therefore not included in the NFI. However, using remote sensing they will be identified either as grassland (the underlying vegetation) or as tree cover – depending on the density of the fruit trees. In any case, this type of vegetation contributes to the overall tree cover. The upper left corners of Fig. 3g and h are missing data in the satellite image.

For tile 30VUH the coniferous forest compares reasonably well with the NFI whereas the broadleaved forest overestimates the tree cover. This is likely related to the spectral signature of these trees not being significantly different from their surroundings.

For tile 32VNH there is good qualitative agreement between the satellite-derived map and the NFI. However, the satellite derived image shows regions with somewhat higher amounts of trees compared to the NFI. According to Corine Land Cover this region also contains substantial areas of urban land cover (in particular cities of Aarhus, Silkeborg, Randers and Horsens) and these areas have in the remote sensing picture been classified with substantial woodland cover whereas the NFI does not include those regions. As such, part of the difference is actual trees not included in the NFI whereas another part of the difference is spectral confusion between trees and green fields/grass.

The summary statistics for the present study and the respective NFI plus the statistics for the studies by Kempeneers et al. (2011) and Hansen et al. (2013) are presented in Table 4, Table 5, Table 6, Table 7. Tile 33VUC is the only image where the present study yields a smaller total tree cover compared with the other datasets. As described above, this is caused by cloudy areas in the image, not included in the accompanying cloud mask, but removed by the NDVI-filter of the present algorithm. As seen from Section 2.1, the present algorithm does not distinguish between clouds and non-forest pixels. The reason is that this distinction is complicated and thus beyond the scope of the present study (see Deng et al. (2019); Li et al. (2019); Sui et al. (2019) for some recent examples). The present approach is designed for cloud free or almost cloud free images and is designed with computational efficiency in mind. Besides that, incorporating multiple images over the same area in a subsequent study is expected, to some extent, to alleviate this problem. Despite this bias, the total tree cover is quite close to the other estimates, which indicates that the remaining areas have a larger tree coverage than previously thought. Part of this tree cover is technically not accounted for in the NFI as the land use is either agricultural (e.g. orchards), urban (e.g. low density residential) or recreational (summer cottages).

**Table 4:** Summary statistics for tile 33VUC.

Tile:	33VUC		
Dataset:	Broadleaved Trees (km^2^):	Coniferous Trees (km^2^):	Total tree cover (km^2^):
Present study	866	1309	2175
Kempeneers et al. (2011)	1100	1422	2522
National forest inventory	–	–	2676
Hansen et al. (2013)	–	–	2334

**Table 5:** Summary statistics for tile 30UWC.

Tile:	30UWC					
Dataset:	Broadleaved Trees (km^2^):		Coniferous Trees (km^2^):		Total tree cover (km^2^):	
Date:	19.07.2016	15.08.2016	19.07.2016	15.08.2016	19.07.2016	15.08.2016
Present study	1047	666	101	320	1148	986
Kempeneers et al. (2011)	806	737	103	86	909	823
National forest inventory	796	738	208	175	1004	913
Hansen et al. (2013)	–		–		868	783

**Table 6:** Summary statistics for tile 30VUH.

Tile:	30VUH		
Dataset:	Broadleaved Trees (km^2^):	Coniferous Trees (km^2^):	Total tree cover (km^2^):
Present study	1330	875	2204
Kempeneers et al. (2011)	94	1194	1288
National forest inventory	357	1078	1435
Hansen et al. (2013)	–	–	1488

**Table 7:** Summary statistics for tile 32VNH.

Tile:	32VNH		
Dataset:	Broadleaved Trees (km^2^):	Coniferous Trees (km^2^):	Total tree cover (km^2^):
Present study	1892	496	2387
Kempeneers et al. (2011)	237	798	1035
National forest inventory	–	–	1673
Hansen et al. (2013)	–	–	1149

For the remaining images, the relative difference between the tree cover area of the present study and the previous studies is between 8% and 79%. As can also be seen, there is a large variation between the previous estimates of the tree cover for the respective image, which contributes to the large variation in the relative difference between the previous estimates and the present study. For tile 33UWC the image dated 15.08.2016 always has a smaller tree cover compared with the image 15.07.2016 due to the smaller area covered by the satellite on this particular day. As can be seen, even though the total area does not change much, the distribution between broadleaved and coniferous trees changes significantly – a result also seen in Fig. 3e–h. The image from 19.07.2016 has a larger cover of broadleaved trees and a smaller cover of coniferous trees compared with the national forest inventory and vice versa for the image from 15.08.2016. The correct result is probably somewhere between the two estimates, which underlines that temporal averaging or other approaches that utilize several images in order to create accurate tree maps would yield a higher accuracy.

For tile 33 VUH the cover of coniferous trees is in reasonably agreement with the national forest inventory, whereas the cover of broadleaved trees is much larger. A visual inspection of the image reveals that the area of broadleaved trees in Corine Land Cover in this image is much smaller than the area of coniferous trees in Corine Land Cover. This means that there is a larger probability that clouds and other artefacts can influence the training data and thus introduce noise in the labelling procedure. This is also seen in that approximately six times as many pixels are used for the labelling of conifer trees compared with the labelling of broadleaved trees. Future work should aim at reducing this effect.

For 32VNH the present algorithm also finds a considerably larger tree cover compared with the previous studies, but given the large variation in the previous estimates, it is difficult to conclude on the validity of this estimate. However, it is known that this particular area contains a substantial amount of land cover that technically is not part of the national forest inventories (e.g. urban land) and that the tree density in these areas requires at least 10 m spatial resolution in order to be accurately mapped (Uuemaa et al., 2013). This suggests that the true tree cover in those regions likely to be better mapped with the Sentinel-2 satellite compared to previous estimates.

### 3.3 Accuracy assessment

The results of the accuracy assessment for the primary land cover class for tile 30UWC can be found in Table 8 for three categories and in Table 10 for two categories. As can be seen it was not possible to manually classify 58 pixels. Since this corresponds to approximately 5% of the data this is not assumed to influence the results, which can also be seen on the low standard errors on all the accuracies. The overall accuracy for three classes is 90% with a standard error of 1.35%. Comparing this to the commonly used accept criterion of 85%, the accuracy of the map is high, even though this acceptance criterion has been questioned (Foody, 2006). The producer’s and user’s accuracies are quite low for coniferous trees, even though the area comparisons are quite close to each other. It is natural that the accuracy of the map will be better between the no trees category and the two tree categories, compared to between the two tree categories due to the spectral similarity between different types of trees. The significant fraction mapped as broadleaved trees being no trees is most likely green fields which have a similar spectral signature. A part of the pixels mapped as coniferous trees being broadleaved trees is due to shadows e.g. at forest roads or forest edges, where the shadows cause the trees to appear darker and thus fall in the coniferous category. Future work should aim at reducing this effect. When classifying the map in two classes (trees/no trees) the percent correctly classified is 90% again being above the accept criterion. It is noteworthy, that even though the present map has reported substantially more tree cover compared to previous maps, as shown in Section 3.2, the accuracy assessment indicates that the tree cover based on the reference data is actually substantially higher – especially for broadleaved trees. The actual tree cover might therefore be substantially higher.

**Table 8:** Accuracy assessment for tile 30UWC in percentages of area. The table includes the user’s accuracy (User) and the producer’s accuracy (Prod), standard errors are presented in parentheses along with the number of pixels in each category (n). Estimated overall accuracy is 89.97% with a standard error of 1.35%.

	Map					
Reference	No trees	Broadleaved	Coniferous	Total	Prod (SE)	n
No trees	82.89	1.98	0.06	84.92 (1.87)	97.60(0.25)	388
Broadleaved	6.29	6.73	0.59	16.61 (1.21)	49.42 (4.65)	433
Coniferous	0.82	0.30	0.35	1.46 (0.45)	23.80 (8.15)	120
Total	90.00	9.00	1.00	100.00		
User (SE)	92.10 (1.49)	74.75 (2.49)	34.85 (2.72)			
N	329	305	307			941

The results of the accuracy assessment for three land cover classes for tile 30TWN are shown in Table 9 and for two classes in Table 11. The overall accuracy is 83.43% with a standard error of 1.64% for three classes, and as such, a little bit lower than for tile 30UWC. This is expected, since tile 30UWC has been part of the development process for the algorithm. In particular the separation between broadleaved and coniferous trees is better for this tile. The accuracy for the no trees category is slightly lower compared to tile 30UWC. A part of this can be explained by 41 pixels where the manual interpreter could not determine whether the pixel was showing orchards of young trees or fruit bushes, where the tree map has classified it as no trees. This is one of the explanations why the number of unclassified pixels is slightly higher for this image, and the standard error therefore slightly larger. As stated in Section 2.4.1, pixels for this analysis were only sampled within a 10 km radius of the four cities in the image. This means that the actual accuracy for the entire image is likely to be higher, since the land cover will be more homogeneous in the rural areas. Reducing the number of classes to two (trees/no trees) gives an overall accuracy of 85.43% with a standard error of 1.87%. The phenomenon that the area of tree cover estimated from the reference data is substantially higher than the mapped area is likewise found for this tile.

**Table 9:** Accuracy assessment for tile 30TWN in percentages of area. The table includes the user’s accuracy (User) and the producer’s accuracy (Prod), standard errors are presented in parentheses along with the number of pixels in each category (n). Estimated overall accuracy is 83.43% with a standard error of 1.64%.

	Map					
Reference	No trees	Broadleaved	Coniferous	Total:	Prod:	n
No trees	76.11	0.80	0.39	77.29 (2.23)	98.47 (0.34)	317
Broadleaved	10.13	4.40	2.20	16.72 (1.58)	36.31 (2.66)	418
Coniferous	2.76	0.27	2.92	5.95 (0.86)	49.03 (7.69)	191
Total	89.00	5.47	5.50	100.00		
User	85.52 (2.07)	80.50 (2.21)	53.04 (2.83)			
N	290	323	313			926

**Table 10:** Accuracy assessment for tile 30UWC in percentages of area for two categories. The table includes the user’s accuracy (User) and the producer’s accuracy (Prod), standard errors are presented in parentheses along with the number of pixels in each category (n). Estimated overall accuracy is 90.43% with a standard error of 1.38%.

	Map				
Reference	No trees	Trees	Total:	Prod:	n
No trees	82.39	1.96	84.35 (1.89)	97.68 (0.18)	388
Trees	7.61	8.04	16.65 (1.32)	51.37 (4.02)	579
Total	90.00	10.00	100.00		
User	91.54 (1.53)	80.41 (1.57)			
N	331	636			967

**Table 11:** Accuracy assessment for tile 30TWN in percentages of area for two categories. The table includes the user’s accuracy (User) and the producer’s accuracy (Prod), standard errors are presented in parentheses along with the number of pixels in each category (n). Estimated overall accuracy is 85.43% with a standard error of 1.87%.

	Map				
Reference	No trees	Trees	Total:	Prod:	n
No trees	75.59	1.16	76.74 (2.24)	98.49 (0.30)	317
Trees	13.41	9.84	23.26 (1.78)	42.33 (1.97)	632
Total	89.00	11.00	100.00		
User	84.93 (2.09)	89.49 (1.20)			
N	292	657			949

## 4 Conclusion

Tree maps with high thematic accuracy can be produced from Sentinel-2. The high spatial resolution of this satellite means that a larger tree cover is generally found compared with previous estimates (on average 36%), for the five Sentinel-2 tiles in the present study and in particular a large tree cover is found in regions officially classified as urban landscapes. The performance of the present map compared to the respective national forest inventory does not depend on the number of bands included in the analysis, but on the choice of bands, with the band combination 2, 3, 6 and 12 as the best performing combination in the present study. Likewise, the difference in performance for the individual band combination is larger for the different images compared with between the band combinations. With a few exceptions, the present tree map agrees well with the corresponding national forest inventory, and add to this the non NFI tree resource. This non NFI resource can in some regions be substantial. The thematic accuracy, for the two tiles where accuracy assessment was performed, was above or close to the commonly applied 85% threshold for three land cover classes (non-forest, broadleaved trees, and coniferous trees) at a resolution of 10 m × 10 m.

## Declaration of Competing Interest

None.

## Appendix A Using Globcover as training data

To test the sensitivity of the map to the use of Corine Land Cover as training data for the labelling procedure and to test the applicability of the procedure outside Europe, the tile 30UWC was classified using Globcover as training data. Tile 30UWC was selected since the thematic accuracy of the produced tree map was known for this tile. The coniferous trees were represented by class 70 *closed needle leaved evergreen forest*. A natural choice for broadleaved trees would be class 50 *closed broadleaved deciduous forest* or class 40 c*losed to open broadleaved evergreen or semi-deciduous forest.* However, tile 30UWC contains hardly any pixels classified as class 50 and none classified as class 40. Instead, class 110 *Mosaic Forest-Shrubland/Grassland* was used for broadleaved forest, knowing well that this will introduce considerable noise in the training data.

The classification algorithm was run as described in Section 2.1 with the exception that the distribution for the NDVI filter (step 5) was only based on the polygons classified as class 70 in Globcover, since class 110 contained significant non-forest elements.

A visual inspection of the resulting map shows good agreement between the two maps, with the map based on Globcover resulting in 639 km^2^ broadleaved trees and 236 km^2^ conifer trees. The map using Globcover as training is thus probably reasonably accurate with respect to coniferous trees, but underestimates the number of broadleaved trees. These numbers compare well with the figures in Table 5, which is further underlined by the wall-to-wall kappa coefficient between the map based on Globcover and the map based on Corine Land Cover being 0.58. In this way, the applicability of Globcover as training data is shown.
