# Supplementary material for: Tree cover mapping based on Sentinel-2 images demonstrate high thematic accuracy in Europe
Source: Int J Appl Earth Obs Geoinf. 2020 Feb;84:101947. doi: 10.1016/j.jag.2019.101947 (PMC8804947; doi:10.1016/j.jag.2019.101947)
Supplement: Supplementary file 1 [file mmc1.docx]

# Supplementary material for Tree Cover Mapping Based on Sentinel-2 Images Demonstrate High Thematic Accuracy in Europe

Thor-Bjørn Ottosen^a,*^, Geoffrey Petch^a^, Mary Hanson^a^, Carsten Skjøth^a^

^a^ School of Science and The Environment, University of Worcester, Worcester, UK

*Corresponding author, E-mail address: t.ottosen@surrey.ac.uk (T.-B. Ottosen)

## 1. RGB images of the Sentinel-2 tiles


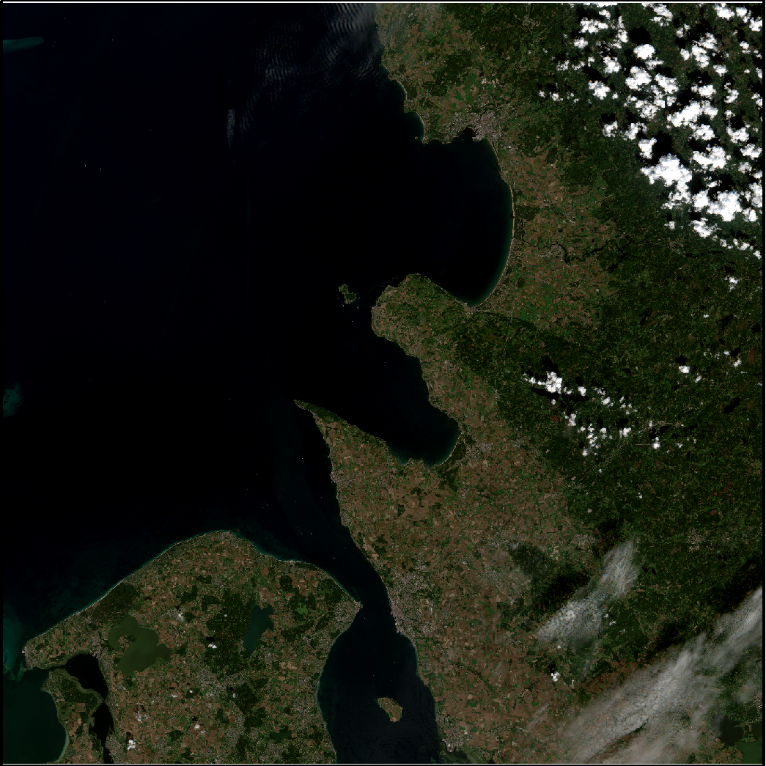

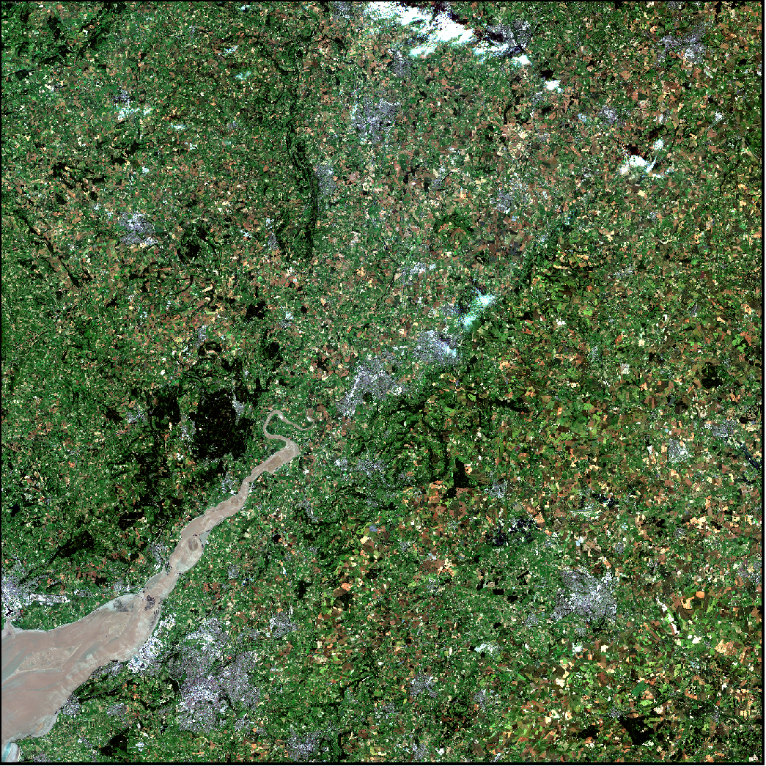


a) RGB image of tile 33VUC b) RGB Image of tile 30UWC from 19.07.2016


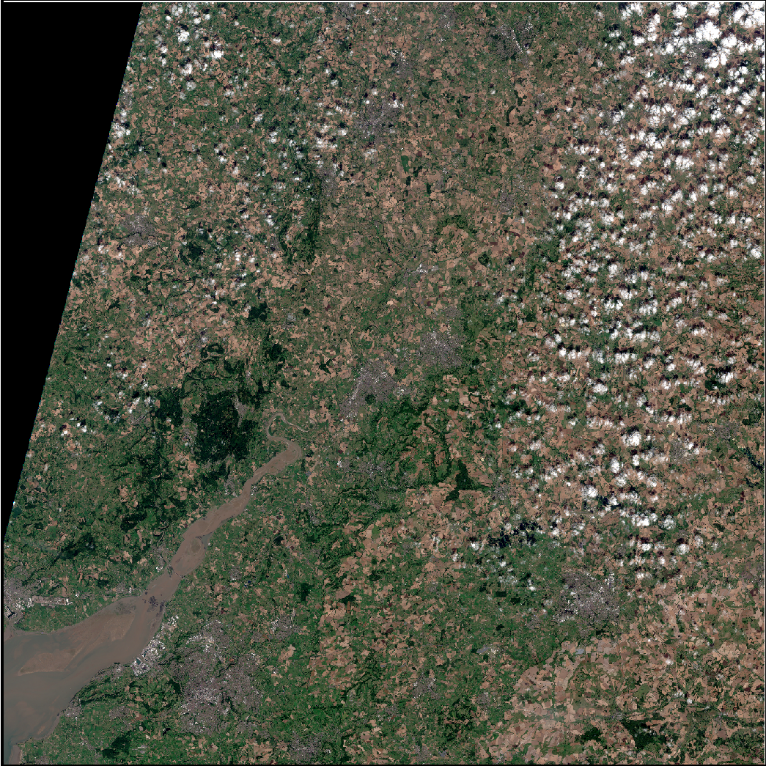

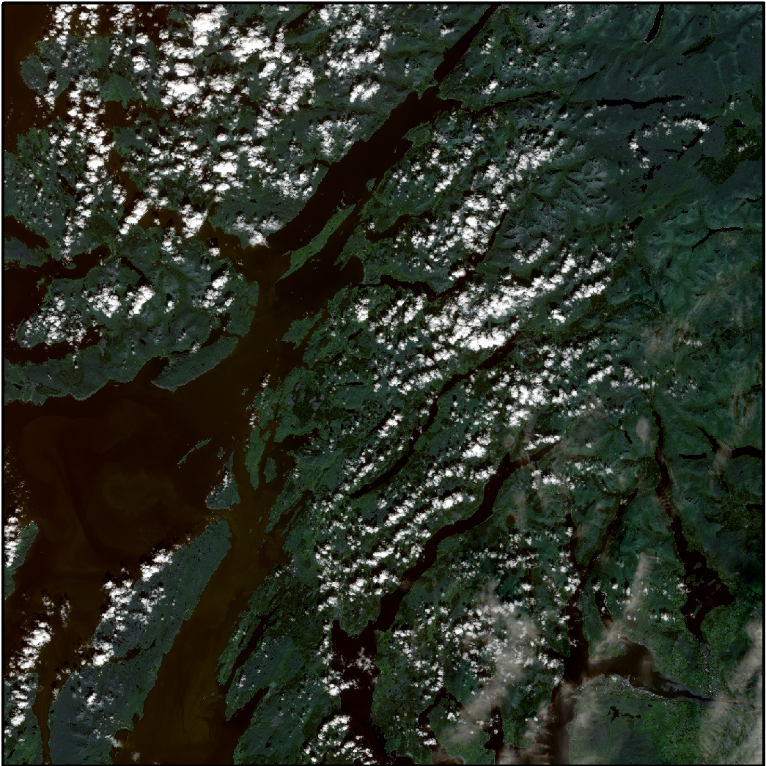


c) RGB image of tile 30UWC from 15.08.2016 d) RGB image of tile 30 VUH


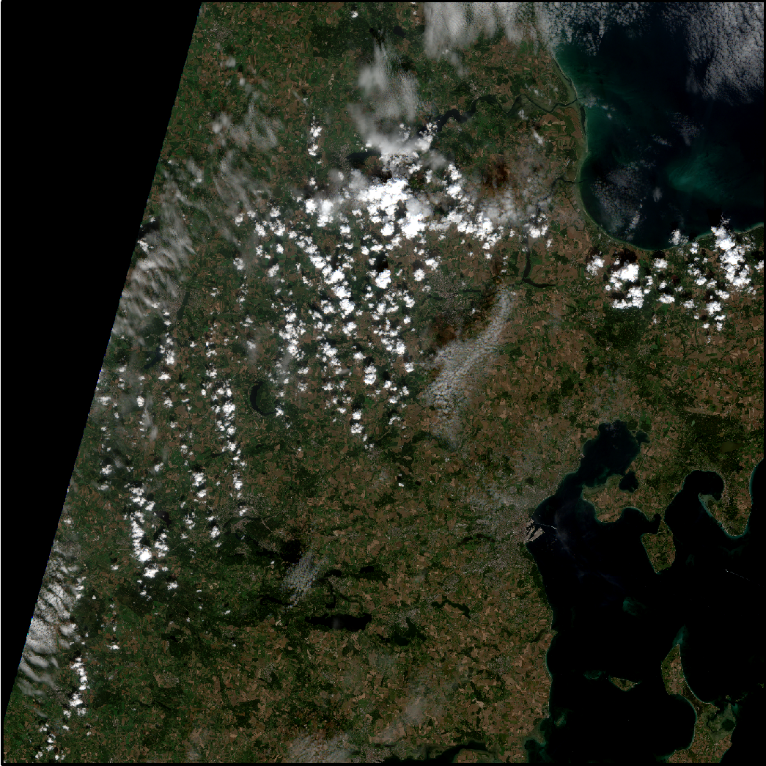


e) RGB Image of tile 32VNH.

Figure 1 RGB images of the five Sentinel-2 images analysed in the present study.

## 2. Map of the location of the tiles

Figure 2 Location of the tiles used in the present study.

## 3. Elevation map of tile 30UWC


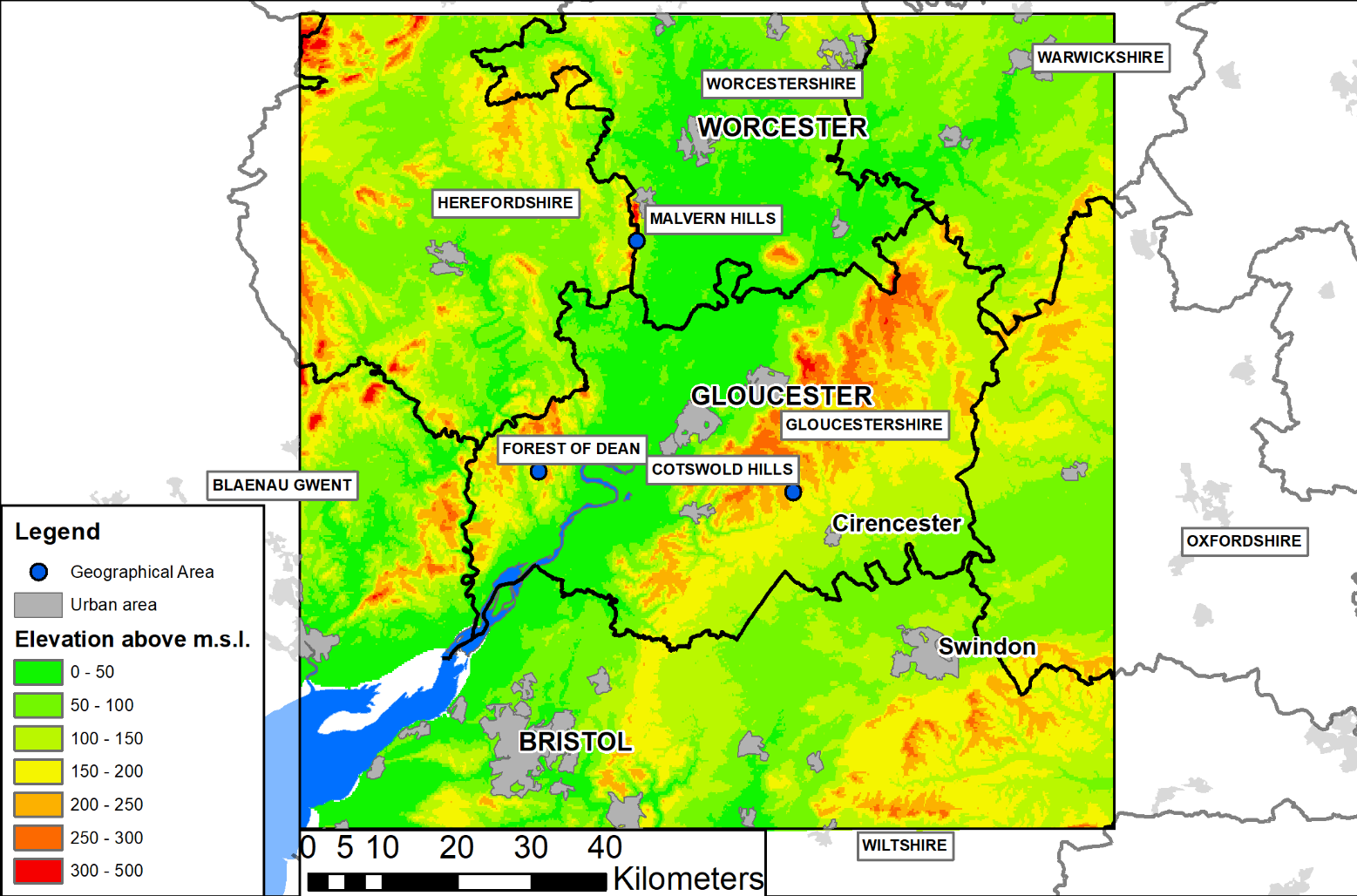


Figure 3 Elevation map of the tile 30UWC. Data sources: Counties, Urban areas, Surface water (CLC), elevation (the SRTM mission) (Reuter et al., 2007).


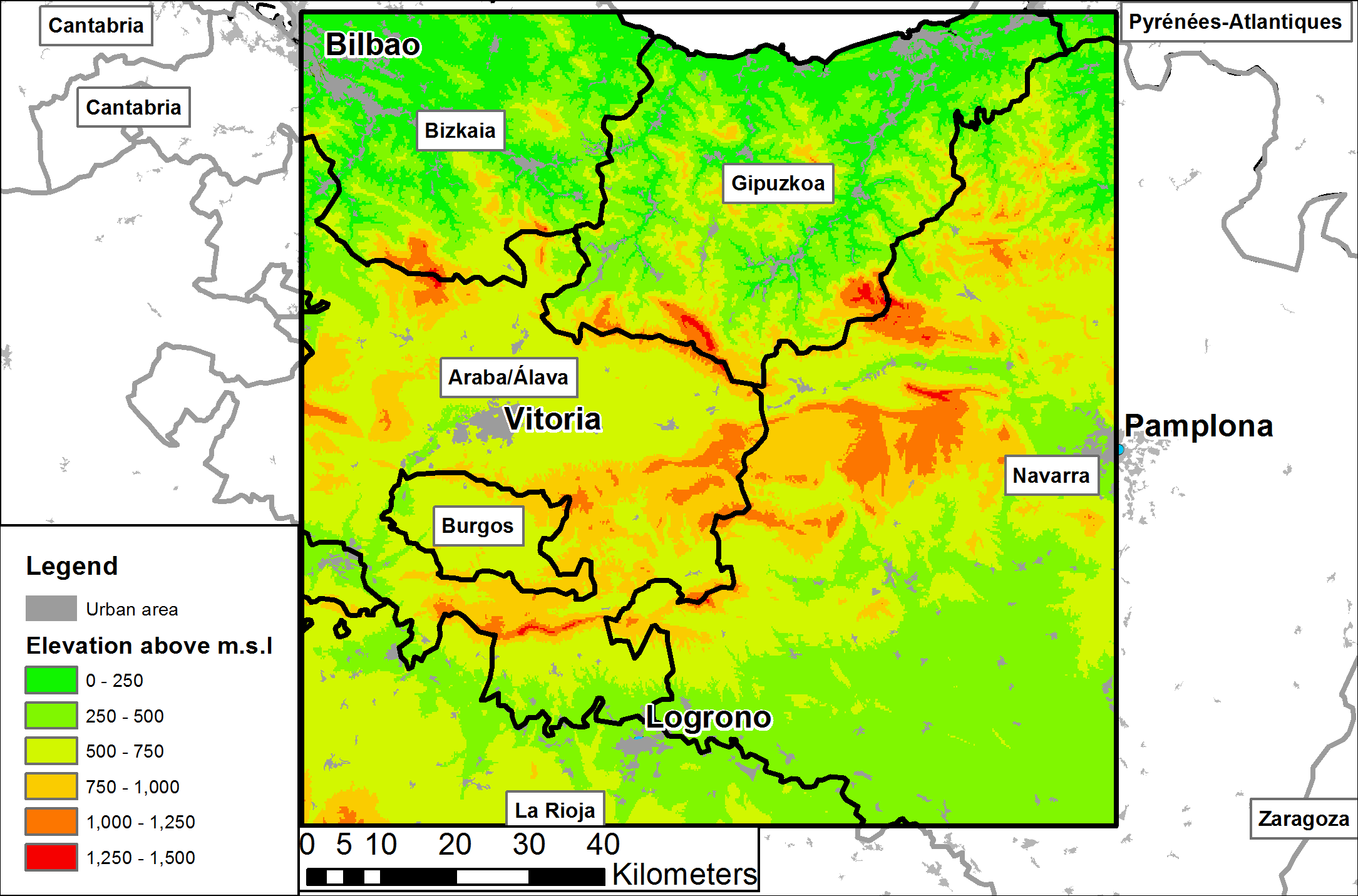


Figure 4 Elevation map of the tile 30TWN. Data sources: Counties (Eurostat NUTS, https://ec.europa.eu/eurostat/web/gisco/geodata/reference-data/administrative-units-statistical-units/nuts), Urban areas (Bossard et al. 1994), elevation (the SRTM mission)(Reuter et al. 2007).
